# Meniscal Root Fixation Method for Meniscus Transplantation: is There a Superior Technique?

**DOI:** 10.1007/s12178-026-10007-0

**Published:** 2026-02-06

**Authors:** Romed P. Vieider, Jasmine Wang, Jacob Hartline, Shu Watanabe, Karina Dias, Joseph Euringer, Seth L. Sherman, Volker Musahl

**Affiliations:** 1 Department of Orthopaedic Surgery, UPMC Freddie Fu Sports Medicine Center, University of Pittsburgh, Pittsburgh, PA 15203 USA; 2 Department of Sports Orthopaedics, TUM University Hospital, Technical University of Munich, Munich, Germany; 3 Department of Orthopaedic Surgery, Kobe University Graduate School of Medicine, 7-5-1 Kusunoki-cho, Chuo-ku, Kobe, Hyogo 650-0017 Japan; 4 Department of Orthopaedic Surgery, School of Medicine, Stanford University, Redwood City, CA USA

**Keywords:** Meniscal allograft transplant, Root fixation, Meniscus, Bone plug, Arthroscopy

## Abstract

**Purpose:**

Meniscal allograft transplantation (MAT) is a well-established option for young, active patients with symptomatic functional meniscal deficiency, aiming to restore knee function and delay the progression of degenerative changes. This review provides an overview of available root fixation techniques and contextualizes their clinical outcomes to help surgeons select the most suitable method for individual patients.

**Recent Findings:**

Multiple fixation approaches, including all–soft-tissue, bony fixation (bone plug or bone bridge), and hybrid techniques have demonstrated significant improvements in patient-reported outcomes and pain reduction. Bony fixation may reduce or equalize graft extrusion relative to soft-tissue methods, though the clinical significance of extrusion remains uncertain. While functional outcomes and long-term survivorship appear similar across techniques, soft-tissue fixation may carry a slightly higher rate of graft tears and complications. Long-term follow-up reveals that osteoarthritis progression and graft degeneration can still occur. Hybrid fixation techniques combine bony and all-soft-tissue root fixation to leverage the advantages of both methods. It is important to address related conditions such as bone malalignment and ligament instability.

**Summary:**

Overall, MAT provides durable improvements in knee function and quality of life regardless of fixation method. Optimal results depend not only on the fixation technique but also on addressing concomitant pathologies, such as ligamentous instability and malalignment, with osteotomy playing a key role in protecting the graft when necessary. As no single fixation method has proven superior, further research is required to clarify the ideal strategy and the true clinical impact of graft extrusion.

## Introduction

The menisci in the knee joint are elastic tissues that play a crucial role in transmitting biomechanical forces effectively. As an example, almost 50% of the load on the medial joint is transmitted through the medial meniscus [14]. Meniscal lesions are one of the most common reasons for pain in the knee joint and are commonly repaired with different surgical techniques. For young patients with functional meniscus deficiency due to large meniscectomy or irreparable meniscal tears, meniscal allograft transplantation (MAT) is a potential treatment option [16, 43]. The principle of this procedure is to restore tibiofemoral load distribution when the native meniscus can no longer do so. The indications for MAT are not clearly defined; however, there is agreement in the literature that MAT transplantation is targeted for individuals < 50 years with symptomatic meniscal deficiency. If high-grade cartilage wear, joint instability, or limb malalignment are present, they should be treated simultaneously with the MAT [8, 16, 17]. Long-term results of MAT show promising graft survival and sustained functional improvement [4, 38]. The most common cause of failure is posterior root tear of the allograft in up to 40% of MAT failures [50]. Several techniques for MAT fixation have been described [3–5, 20, 32, 47]. The MAT root fixation techniques are mainly classified into two categories including bony or all soft tissue fixation. Bony fixation profits from bone-on-bone healing and compared to soft tissue fixation resulted in less extrusion but may be more invasive and challenging when the MAT graft doesn’t perfectly fit the joint [10]. There remains a paucity of data on whether MAT extrusion is clinically relevant. All soft tissue fixation results in equivalent clinical and functional outcomes as bony fixation and is technically less invasive and easier to implant [4]. The prevalence of either fixation technique reported in the literature is roughly equal [38, 50, 53]. Given the varying perspectives in MAT root fixation, the purpose of this review is to provide a comprehensive review on different techniques and advances in MAT root fixation. Furthermore, differences in clinical and functional outcomes will be provided.

### Evolution of MAT - Surgery

The first publication describing MAT appeared in 1987, where the authors used an open approach [37]. Arthroscopy was incorporated shortly after, and minimally invasive procedures with mini-open approaches were developed to utilize MAT [22, 36]. Consequently, arthroscopic MAT techniques have been described using a simple extension of the standard arthroscopy portals. Depending on the MAT fixation method, a small tibial incision may be added for transtibial tunnel drilling and fixation [20, 46, 53]. Technological advances have promoted minimally invasive approaches, especially improvements in root and capsular fixation methods. Initially, direct suturing of the meniscus to the capsule was employed. However, techniques have evolved to include arthroscopic-assisted inside-out suture fixation and all-inside fixation methods, which are reported to have similar outcomes and shorter operative times [2, 36, 37, 53].

The fixation of the anterior and posterior roots facilitates MAT function to fulfill its biomechanical role by restraining hoop stress and optimizing the distribution of tibiofemoral load [14]. The posterior root of the MAT is particularly significant because the most frequent reason for revision surgery is failure of the MAT posterior root. Various strategies for MAT root fixation exist.

The posterior root of the MAT warrants particular attention, as posterior root failure is the most frequent cause of revision surgery [12, 41, 48]. Although various root fixation strategies exist anatomic positioning appears to be the critical factor determining success, irrespective of the specific technique used [2, 13, 33, 46, 53].

Technical adjuncts such as native root remnant preservation, deep medial collateral ligament trephination, and reverse notchplasty can facilitate anatomic placement by improving visualization of the insertion site and optimizing instrument access.

### Bony Fixation

#### Bone-Plug Technique

In the bone plug technique, the MAT preserves the native meniscus root insertions into the bone. Two separate bone plugs, each attached to the anterior and posterior meniscal roots, are used to secure the allograft in its anatomical position. Bone plug fixation may be preferable for medial MAT due to greater distance and variability of the root insertion anatomy [10, 35, 40]. Additionally, medial meniscal root insertions are more difficult to consistently replicate, and a non-anatomic position can increase extrusion of the transplanted meniscus [24, 26]. The clinical relevance of MAT extrusion is not fully understood, but efforts are ongoing to improve the accurate reconstruction of the native medial meniscal anatomy [35]. Various techniques for securing bone plugs into the bone have been described, with interference screw fixation being the most common method, but the use of all suture anchors and transtibial suspensory button systems has been proposed as well [23, 39].

### Bone-Bridge Technique

In contrast to harvesting two separate bone plugs, the bony bridge technique aims to harvest one single piece of bone that contains both the anterior and posterior root insertions [4, 24]. This technique may be particularly well-suited for lateral MAT, where the anatomical configuration differs significantly from the medial side: the lateral meniscal roots are positioned closer together with a more anterior-posterior orientation, whereas the medial meniscal roots are spaced further apart with a more oblique trajectory that overlaps the ACL footprint [14]. Bone bridge techniques have been shown to recreate root insertions in the lateral meniscus within 5 mm of the native anatomy, and when used in lateral MATs, they more closely restore native biomechanics compared to bone-plug techniques [6, 25, 52]. However, these advantages must be weighed against the technical demands of the procedure, as more extensive surgical approaches are necessary to facilitate bony bridge MAT implantation compared to minimally invasive bone-plug fixation techniques.

### All Soft Tissue

The MAT graft is typically prepared using non-absorbable sutures, which are placed in the anterior and posterior horns in a modified Burnell-stitch fashion. Transtibial tunnels in the anterior and posterior meniscal root footprints are created. The tunnels are used to pass through the sutures which are secured at the tibial cortex. Multiple fixation options have been described, including transtibial pull-out sutures tied at the tibial cortex [18, 36, 49], suspensory fixation with a cortical button [4, 44], and tibial anchor fixation [47]. These methods provide secure root fixation and are widely used for both medial and lateral MAT [1, 21].

### Hybrid Techniques

**Fig. 1:**
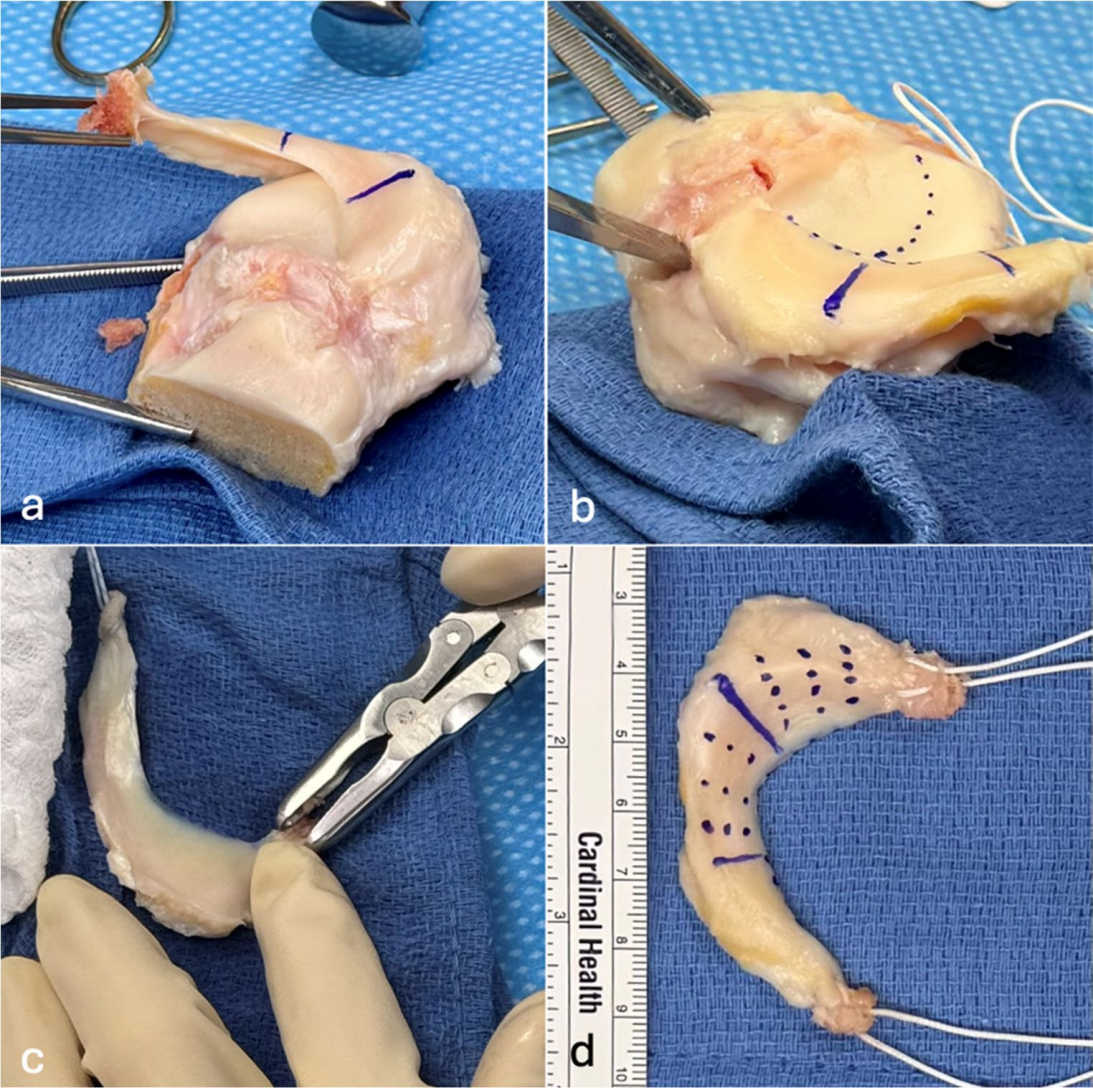
Left sided Medial hemi plateau of a fresh frozen meniscal allograft transplant (MAT). **a** The three different anatomical parts (anterior horn, midbody, posterior horn) of the MAT were marked and the anterior root was harvested with bone attached to it. **b** The posterior root is harvested with the osteotome. The dots highlight the coverage of the tibial joint surface by the meniscus meniscus. **c** The bony sliver attached to the root is then thinned to form a thin bony surface that stay attached to the root. **d** A braided mattress suture is passed through each MAT root and bony sliver. Once the MAT is positioned intra-articularly, dotted lines indicate the positions for the sutures, aiding in the accurate placement of the sutures within the joint

To combine the benefits of bony fixation (bone-to-bone healing) and all–soft-tissue fixation (greater flexibility and arthroscopic feasibility), the senior author employs a hybrid technique. The hybrid technique involves the use of a fresh-frozen hemi-plateau allograft with a 1–2 mm bone sliver preserved at each meniscal root (Fig. 1). This small bone fragment allows bone-to-bone healing while maintaining the technical facility of soft-tissue fixation. During MAT graft preparation, an osteotome is used to remove each root while leaving the bone attached. Then, the bony parts are trimmed, leaving a small bony sliver at the MAT anterior and posterior roots attached. A braided mattress suture is placed through each MAT root and bony sliver for later fixation, as it is used in most described soft tissue fixation methods [13, 18] (Fig. 1).

**Fig. 2:**
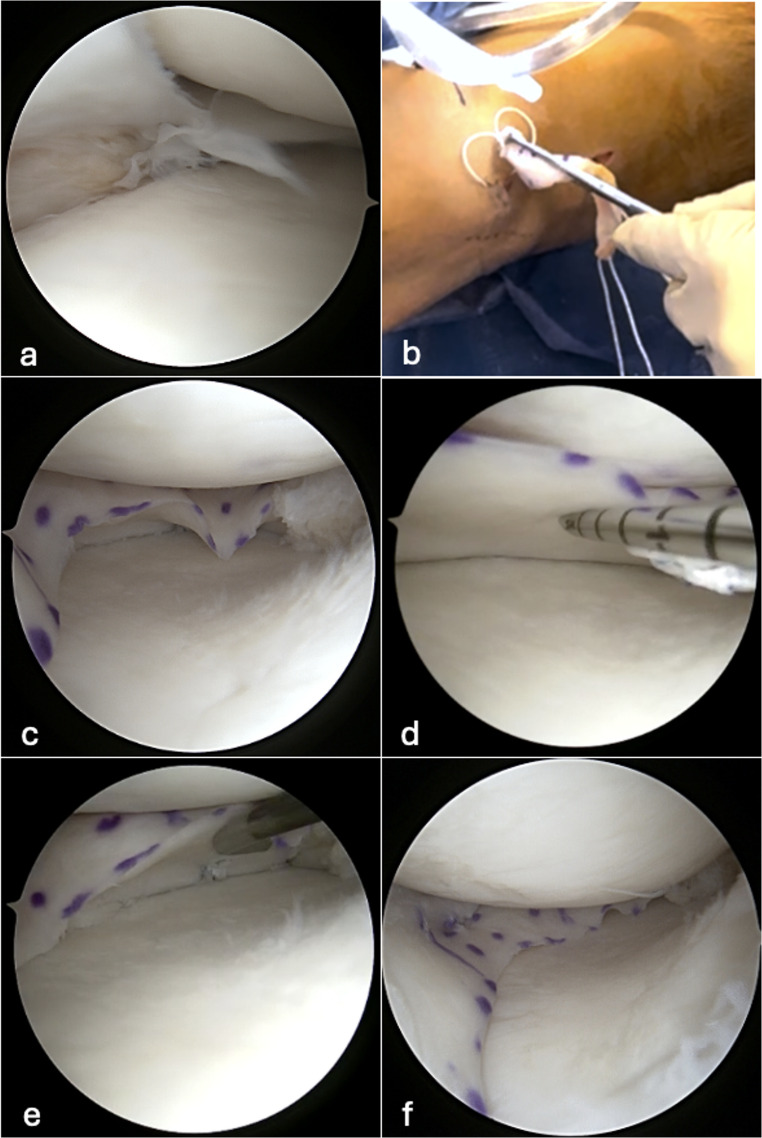
Sequential illustration of meniscal allograft transplantation (MAT) implantation. **a** Intraoperative arthroscopic image of the medial compartment of a left knee with meniscal insufficiency. The native meniscus is extruded, and a radial midbody tear is visible. **b** The MAT is introduced through the medial arthroscopic portal using a forceps. **c** The MAT is positioned and fixated at the posterior root. **d** Peripheral fixation begins at the transition between the midbody and posterior horn. An all-inside suture is placed on the inferior surface near the graft base. **e** The posterior horn and midbody are secured with peripheral sutures, which are visible when the graft is elevated with an arthroscopic probe. **f** Final configuration and positioning of the MAT

During arthroscopy, the remaining native meniscus is trimmed to a 2–3 mm stable rim. The posterior root insertion is prepared and decorticated with an arthroscopic curette to provide a bleeding surface, then a tibial tunnel is drilled at the anatomic posterior root site, and the MAT graft is introduced with an arthroscopic grasper through the dilated anteromedial or anterolateral portal for medial and lateral MAT, respectively. All-inside vertical mattress sutures are placed from posterior to anterior to secure the graft to the capsule (Fig. 2). The anterior root insertion is prepared and decorticated in the same manner as the posterior root insertion, and the second tibial tunnel is drilled aiming at the insertion of the anterior meniscal root. Both posterior and anterior root sutures can be fixed either together or separately over cortical buttons. This hybrid fixation technique combines the soft-tissue flexibility of a soft-tissue anchor with the bone-to-bone healing of a rigid fixation device for enhanced graft integration.

#### Peripheral fixation of the meniscus allograft transplant

**Fig. 3:**
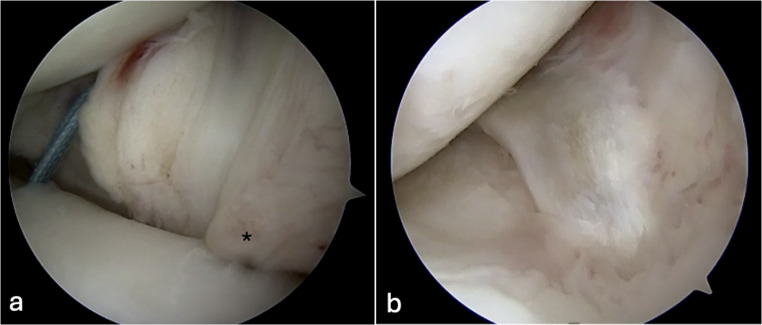
Arthroscopic visualization of lateral capsulodesis in a left knee. **a** Two knotless suture anchors have been inserted at the lateral tibial margin of the plateau. The posterior suture (blue) is visible in its pre-tensioned state, while the anterior suture is obscured by the completed capsulodesis (*), which has already been tensioned and advanced to the tibial attachment site

Reinforcement sutures are typically placed along the MAT periphery using inside-out, outside-in, or all-inside suture techniques to secure the graft to the capsule and native meniscal rim, enhancing stability and promoting biological integration (Fig. 3) [2, 7, 28, 36, 45]. The posterior horn fixation is critical and peripheral sutures placed through an accessory posteromedial portal have been described to restore hoop stress while minimizing neurovascular risk [36]. While biomechanical studies suggest improved initial stability, clinical outcome data remain limited [3, 45]. Capsulodesis and centralization procedures may further enhance graft containment [34]. To optimize meniscotibial stability, the authors suggest compartment-specific capsular augmentation techniques can performed prior to MAT implantation. On the lateral side, a lateral capsulodesis may be completed before graft insertion, advancing the lateral capsule to the tibia using knotless anchors placed through a small iliotibial band-splitting incision (Fig. 3). This approach addresses lateral meniscotibial instability without creating excessive constraint. On the medial side, meniscotibial stability is restored using tape sutures positioned at the junction of the mid-meniscus with both the anterior and posterior horns, incorporating the native meniscal remnant and capsule, and securing these constructs to the tibial bone with small suture anchors. Notably, this medial augmentation technique would likely create excessive constraint if applied to the lateral compartment, highlighting the importance of tailoring capsular repair strategies to the distinct biomechanical demands of each meniscus. Clinical studies have demonstrated that capsulodesis, in combination with suture-only fixation, can significantly reduce graft extrusion and maintain low extrusion rates at mid-term follow-up, with functional outcomes comparable to those of bone fixation techniques [3, 34].

**Fig. 4:**
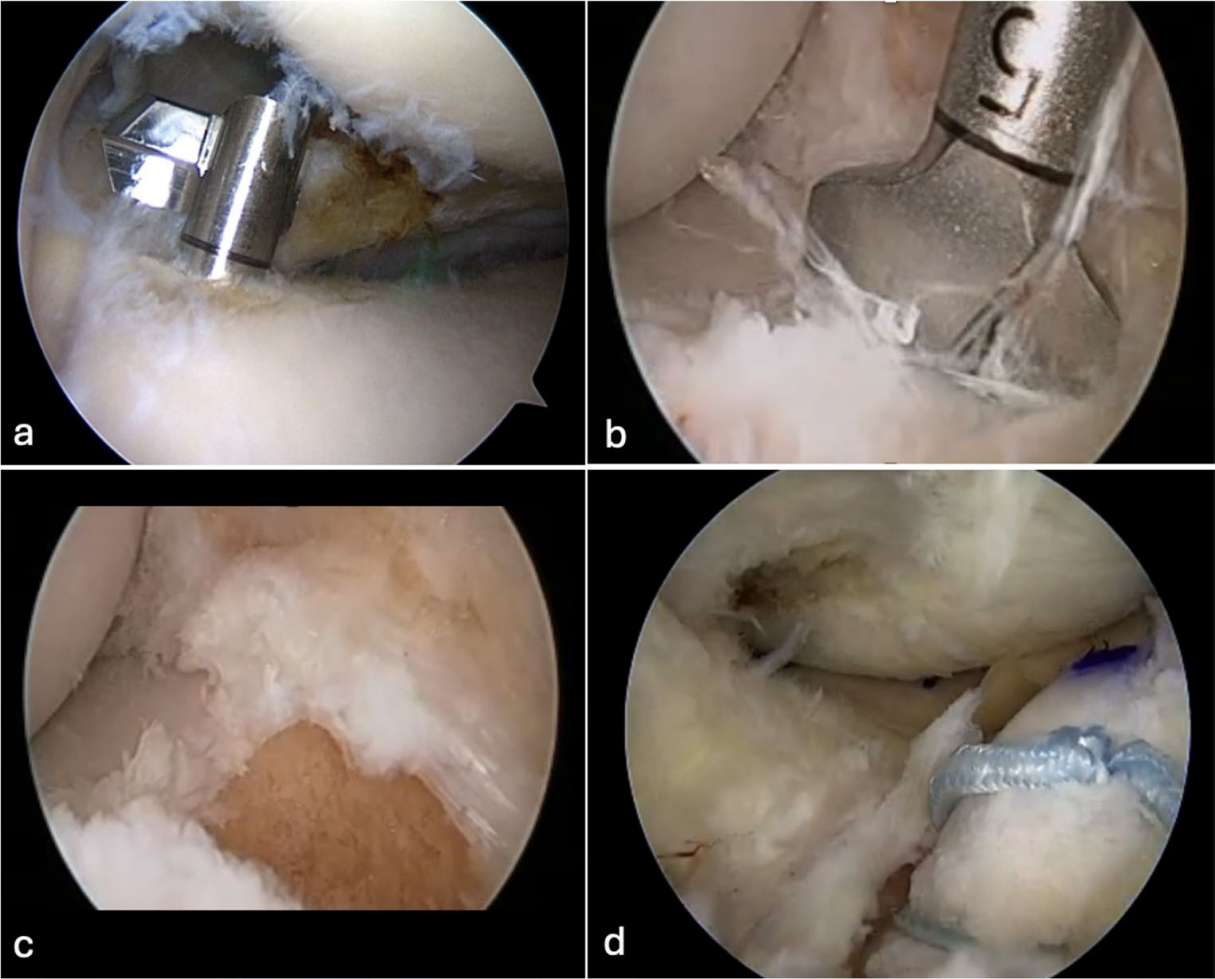
Sequential illustration of meniscal allograft transplantation (MAT) implantation in a right knee. **a** A retrograde drill is used to create a bone socket at the posteromedial footprint of the native meniscus. **b** A 10 mm socket is drill is used from a high anteromedial portal and connected to the anterior cortex using an ACL guide. **c** Compared to posteriorly, the anterior socket is deeper. **d** The deeper socket allows for real-time adjustment of any graft-to-tunnel mismatch by burying the anterior horn deeper into the socket as needed

An additional approach to MAT root fixation that addresses both posterior root security and anterior root adjustability is presented in a second hybrid fixation technique: For the posterior horn, a retrograde drill is utilized to create a socket less than 5 mm in depth (Fig. 4). A small bone plug can then be anchored using a suture anchor placed on the anterior cortex. The anterior horn fixation differs based on the compartment laterality: for medial MAT, a 10 mm socket is drilled from a high anteromedial portal and connected to the anterior cortex using an ACL guide, whereas for lateral MAT, a retrograde drill can be employed to create the anterior socket in close proximity to the ACL footprint. A key advantage of this technique is the use of deeper anterior drilling combined with suspensory fixation on the anterior bone plug, which allows for real-time adjustment of any graft-to-tunnel mismatch by burying the anterior horn deeper into the socket as needed (Fig. 4d). Furthermore, the re-tensionable nature of the anterior suspensory fixation permits peripheral suture placement and graft fixation to be completed first, followed by subsequent re-tensioning of the anterior root to eliminate time-zero meniscal extrusion and optimize graft positioning.

### Future Research

In recent years, additional strategies have been introduced to refine soft tissue fixation. Circumferential suture-tape augmentation has been introduced to recreate hoop stress resistance and reduce graft extrusion [42]. The long-term efficacy of these innovative techniques still needs to be investigated.

### Outcomes

Overall, MAT is a well-established procedure, and numerous studies demonstrate good clinical and functional outcomes, with failure rates ranging between 15% and 40%, largely depending on the duration of reported follow-up. Clinical outcomes display an increase in knee function compared to preoperative. Patients typically return to their preoperative activity levels [5, 18, 38]. Even return to sports rates in recreational and professional sports were reported to be between 45% and 77% [9, 11, 43]. When interpreting outcomes, it is important to consider multiple aspects that may influence clinical and functional outcomes after MAT. Medial versus lateral MATs show differences in their anatomy that should be considered when comparing outcomes. The native medial meniscus has less mobility than the lateral, and this may place more stress on the posterior root, which is the most common site for failure of medial MAT [15, 31]. While root tears remain common causes in lateral MAT, other mechanisms including meniscocapsular separation in the body have been frequently implicated as well [31, 50]. Multiple studies have also demonstrated higher rates of MAT extrusion in medial versus lateral procedures, although there is not consensus on the clinical importance of extrusion in the coronal plane [27, 29, 51]. Frontal limb alignment is an important consideration when planning MAT. Osteotomies are effective procedures for redistributing the load on the knee joint. Outcomes of alignment-correcting osteotomies, whether combined with MAT or performed in two stages, have demonstrated favorable results, and the rate of MAT failures can be reduced [19] There are multiple definitions of failure described in the literature, such as cutoffs of clinical outcome scores, reoperations because of the MAT, or definitions of the endpoints, such as conversion to total knee arthroplasty or total MAT resection [19, 30, 32, 50]. Survival of the MAT graft was reported to be 74% at 10 years and 60% at 15 years, highlighting that, overall, the procedure is effective [18, 38]. The most common reason for MAT graft failure is posterior MAT root tears, which tend to be more prevalent in suture-only fixation (57% vs. 22% with bone blocks), and are strongly linked to early graft failure [1, 50]. The fixation type of MAT-roots may not be the main determinant of outcome, as other studies have also shown that failure or reoperation rates between bone bridge and soft tissue fixation for lateral MAT do not differ [4]. Long-term outcomes show that all-soft tissue fixation provides significant improvements in pain and function, with survivorship at 7–10 years exceeding 85% in large series [18, 33]. The International Meniscus Reconstruction Experts Forum concludes that both bone-based and soft tissue fixation techniques are both valid, and that choice should depend primarily on surgeon expertise and patient-specific factors [16].

## Conclusion

Meniscal allograft transplantation (MAT) is a treatment option for young patients with post meniscectomy syndrome and relative preservation of the knee. Various MAT root fixation methods exist, primarily differing between bony and all–soft tissue fixation, with hybrid techniques aiming to combine the advantages of both. Long-term survival rates and clinical outcomes are generally satisfactory, regardless of the fixation method used. Although bony fixation methods tend to demonstrate less graft extrusion compared to all–soft tissue fixation clinical outcome is similar. Ultimately, the choice of fixation technique should be individualized according to surgeon’s preference and patient-specific factors.

## Key References

- Grassi A, Lucidi GA, Di Paolo S, Altovino E, Agostinone P, Dal Fabbro G, et al. (2024) Clinical Outcomes of Medial Meniscal Allograft Transplantation With or Without High Tibial Osteotomy: A Case-Control Study Up to 8 Years of Follow-up. Am J Sports Med 52:1813-1819.
  - ○ This study showed that medial meniscal allograft transplantation combined with high tibial osteotomy provides midterm clinical outcomes comparable to isolated transplantation, but carries a slightly higher risk of surgical failure over time. Therefore, limb malalignment is no contraindication for meniscal allograft transplantation.
- Winkler PW, Wagala NN, Hughes JD, Irrgang JJ, Fu FH, Musahl V (2021) Association Between Meniscal Allograft Tears and Early Surgical Meniscal Allograft Failure. Am J Sports Med 49:3302-3311.
  - ○ This study showed that meniscal allograft root tears, particularly common with suture-only fixation and in younger patients, were the predominant graft injury and were strongly associated with early postoperative graft failure and significantly reduced 1-year survival.
- Lee DH, Lee CR, Jeon JH, Kim KA, Bin SI (2015) Graft extrusion in both the coronal and sagittal planes is greater after medial compared with lateral meniscus allograft transplantation but is unrelated to early clinical outcomes. Am J Sports Med 43:213-219.
  - ○ This study showed that graft extrusion, particularly in the medial compartment and at the anterior horn, was consistently greater after medial than lateral meniscal allograft transplantation, yet extrusion in either plane did not correlate with early clinical outcomes.

## Data Availability

No datasets were generated or analysed during the current study.

## Abbreviations

MAT: Meniscal Allograft Transplant
mm: Millimeter
